# Influence of Dry Eye Disease on the Measurement Repeatability of Corneal Curvature Radius and Axial Length in Patients with Cataract

**DOI:** 10.3390/jcm11030710

**Published:** 2022-01-28

**Authors:** Takahiro Hiraoka, Hiroki Asano, Tomohiro Ogami, Shinichiro Nakano, Yoshifumi Okamoto, Yoshiaki Yamada, Tetsuro Oshika

**Affiliations:** 1 Department of Ophthalmology, Faculty of Medicine, University of Tsukuba, Tsukuba 305-8575, Japan; y-okamoto@md.tsukuba.ac.jp (Y.O.); oshika@eye.ac (T.O.); 2 Department of Ophthalmology, Tsuchiura Kyodo General Hospital Namegata District Medical Center, Tsuchiura 311-3516, Japan; asanoasa8@gmail.com; 3 Department of Ophthalmology, Tsuchiura Kyodo General Hospital, Tsuchiura 300-0028, Japan; 4 Department of Ophthalmology, Ibaraki Seinan Medical Center Hospital, Sakai 306-0433, Japan; ogm@live.jp; 5 Miyakubo Eye Clinic, Maebashi 371-0044, Japan; 6 Department of Ophthalmology, Ryugasaki Saiseikai Hospital, Ryugasaki 301-0854, Japan; s.nakano@ryugasaki-hp.org; 7 Department of Ophthalmology, Hakujuji General Hospital, Kamisu 314-0134, Japan; 8 Department of Ophthalmology, Mito Kyodo General Hospital, Mito 310-0015, Japan; 9 Japan Medical Affairs, Development Management Department, Japan Business, Santen Pharmaceutical Co., Ltd., Osaka 530-8552, Japan; yoshiaki.yamada@santen.com

**Keywords:** dry eye disease, cataract surgery, biometry, repeatability, corneal curvature radius, axial length, unstable tear film, tear film break-up time, corneal epithelial damage

## Abstract

The influence of dry eye disease (DED) on ocular biometric measurements is unclear. We aimed to investigate the effect of DED on the repeatability of ocular biometric measurements in cataract patients. Overall, 114 eyes scheduled for cataract surgery were enrolled. Before surgery, DED parameters including tear film break-up time (BUT), corneal and conjunctival staining scores, and subjective symptoms were examined. Corneal curvature radius and axial length were assessed twice on the same day using IOLMaster-500 (Carl Zeiss Meditec), and the absolute difference between the two measurements was calculated and used as an index of measurement repeatability. The measurement repeatability of the steep meridian of corneal curvature radius was significantly worse in eyes with DED than in those without DED (*p* = 0.044) and was significantly associated with BUT (r = −0.206, *p* = 0.031). The measurement repeatability of axial length was negatively correlated with BUT (r = −0.199, *p* = 0.041) and positively correlated with the corneal staining score (r = 0.253, *p* = 0.009). In conclusion, the measurement repeatability of corneal curvature radius declined in eyes with DED. Shortened BUTs were associated with a deterioration in the measurement repeatability of corneal curvature radius and axial length.

## 1. Introduction

With advances in modern optical instrument technologies, cataract surgery has become a sophisticated surgery and not merely a lens replacement procedure. Presently, patients have higher expectations for accurate refractive outcomes and excellent quality of vision after intraocular lens (IOL) implantation. Thus, ocular biometry for IOL power calculations has become increasingly important in clinical practice to achieve preoperatively intended outcomes [1]. In particular, corneal curvature and axial length measurements are crucial parameters for IOL power calculations.

IOLMaster (Carl Zeiss Meditec AG, Jena, Germany) is the first non-contact optical biometric device, and it is based on partial coherence interferometry (PCI). The accuracy of measurements with the globally used IOLMaster-500 has been well established [2,3,4,5]. Recently, PCI-based biometry has been considered the gold standard for ocular biometry in cataract surgery [6]. However, several studies have shown that an uneven or unstable tear film can produce optical aberrations, which may directly reduce the accuracy and repeatability of these measurements [7,8]. Inaccurate keratometric measurements due to an unstable tear film can be a critical confounding factor in IOL power calculations because the corneal surface is the greatest refractive component, accounting for approximately 70% of the refractive power of the human eye.

Dry eye (DE) is a common disease that causes an unstable tear film with an irregular surface. There are two definitions of DE, as follows: the International Dry Eye WorkShop (DEWS) definition [9] and the Asia Dry Eye Society (ADES) definition [10]. The DEWS definition focuses on inflammation and osmolarity as potential risk factors for DED, whereas the ADES definition simply focuses on instability of the tear film [10]. The effect of tear osmolarity, which is one component of the DEWS definition, on the repeatability of keratometric measurements in patients undergoing cataract surgery has been previously investigated [7]. The hyperosmolar group had a significantly higher variability in the average keratometric readings than the normal osmolarity group [7]. However, the influence of tear osmolarity on the repeatability of the axial length measurements, which is another crucial parameter for IOL power calculations, was not examined in this previous report. Furthermore, no studies have examined the effect of tear break-up time (BUT), a component of the ADES definition and an index of tear stability, on the repeatability of keratometric measurements and axial length measurements. Additionally, the influence of epithelial damage, which is one of the important clinical indices of DE disease (DED), on these repeatabilities remains unknown.

The prevalence of DED increases with age [11]. Miyake et al. investigated the prevalence of DED in patients who underwent age-related cataract surgery and found that more than half of patients were diagnosed with definite or probable DED before cataract surgery [12]. Given the extremely high prevalence, it is important to investigate the influence of DED on optical biometric measurements in patients with cataracts. This study aimed to compare the measurement repeatability of corneal curvature radius and axial length between eyes with and without DED. The relationship between the measurement repeatability and DED parameters was also evaluated.

## 2. Materials and Methods

This study was prospectively conducted at four hospitals (Namegata District Medical Center, Ibaraki Seinan Medical Center Hospital, Ryugasaki Saiseikai Hospital, and Hakujuji General Hospital) in Ibaraki Prefecture, Japan. All participants were provided with a full explanation of the study and they provided written informed consent. This study followed the tenets of the Declaration of Helsinki and was approved by the Review Board of Human Rights and Ethics for Clinical Studies Ethics Review Committee (approval number E2017-05-001). This study was registered with the University Hospital Medical Information Network Clinical Trials Registry (UMIN-CTR; https://www.umin.ac.jp/; accessed on 20 January 2022, Identification number UMIN000029333).

### 2.1. Patients

The inclusion criteria for this study were patients who were scheduled for cataract surgery and aged between 50 and 85 years. Eyes were excluded of patients who received DED treatment within 14 days prior to study entry; who wore contact lenses within 7 days prior to study entry; who were scheduled for ophthalmic surgery other than cataract surgery; with a history of refractive surgery or cataract surgery; with lens opacity of grade 4 or higher in the Emery–Little classification [13]; and with active ocular infection, ocular inflammation, or allergic conjunctivitis.

### 2.2. Examinations

Refractive errors were evaluated using automatic refractometers; the model was not standardized among institutions, but the same instrument was used in each institution throughout the study period. The corneal curvature radius and axial length were measured using IOLMaster-500 (Carl Zeiss Meditec AG, Jena, Germany) at all institutions. A series of assessments that consisted of three sequential measurements of corneal curvature radius and five sequential measurements of axial length were performed. The average corneal curvature radius was calculated automatically, and the weighted average was calculated for axial length; these values were used as a representative value for single-time assessment. These procedures were repeated twice on the same day to minimize environmental effects on dry eyes and each measurement, and the absolute difference between the two assessments was calculated and used as an index of measurement repeatability. Before performing the measurements, the technician staff instructed all patients to blink appropriately and then gaze at the internal fixation target. They were also required not to open their eyelids too widely during the measurements. In addition, no eye drops were applied before the measurement. The second measurement was performed within ten minutes after the first measurement, and all measurements were completed from 2 to 5 p.m. to avoid diurnal changes in dry eye status. The corneal curvature radius was examined for both steep and flat meridians. Lens opacities were observed with a slit-lamp microscope and scored using standard photographs based on the Emery–Little classification.

For DED examinations, subjective symptoms were assessed using a DED-related quality-of-life score questionnaire [14]. The BUT was measured for up to 10 s using a stopwatch or metronome and repeated thrice for each eye after 2 µL of 1% preservative-free sodium fluorescein solution was administered into the lower conjunctival sac using a micropipette. The mean values were calculated and used for the subsequent analyses. Subsequently, the corneal staining score was determined. Similarly, 2 µL of 1% preservative-free lissamine green solution was then administered into the lower conjunctival sac using a micropipette, and the conjunctival staining score was determined. The corneal staining score was evaluated over the whole cornea, and the conjunctival staining score was evaluated at the entire nasal and temporal areas separately; each area was scored on a 0- to 3-point scale (from 0 = no damage to 3 = damage over the entire area) using standardized photographs. In this study, DED was diagnosed separately in the right and left eyes. Staining of the ocular surface, measurement of BUT, and evaluation of staining score were performed by four ophthalmologists (H.A., T.Og., S.N., and Y.O.). Multiple meetings were held among the examiners to standardize the technique of these procedures. The diagnosis of DED was established on the basis of the fulfillment of the following three criteria: (1) a BUT of 5.0 s or less, (2) a total staining score of 3 or more, and (3) the presence of subjective symptoms (frequency score of 1 or more for at least one symptom). Patients who met all of these DED diagnostic criteria including subjective symptoms were enrolled in this study.

### 2.3. Statistical Analysis

The following parameters were compared between the DED and no-DED groups using Welch’s *t*-test: BUT, spherical equivalent refraction, and absolute difference between the first and second assessments for corneal curvature radius and axial length (measurement repeatability). Lens opacity and corneal and conjunctival staining scores were compared between the DED and no-DED groups using Wilcoxon’s rank-sum test. Correlations between DED parameters and measurement repeatability were examined using Pearson’s and Spearman’s correlation analyses. Stepwise multiple regression analysis was also performed to investigate the strength of the associations between several variables and measurement repeatability. The dependent variable was measurement repeatability, and the explanatory variables included the BUT and corneal staining score. All statistical analyses were performed by EPS Corporation (Tokyo, Japan) using SAS version 9.4 (SAS Institute Inc., Cary, NC, USA). Statistical significance was set at *p* < 0.05.

## 3. Results

### 3.1. Demographic and Background Data

Overall, 114 eyes of 69 patients met the inclusion and exclusion criteria and were enrolled in this study. Table 1 and Table 2 show the demographic data and the results of DED parameters, refraction, and lens opacity score in the DED and no-DED groups, respectively. Of the 114 eyes, 67 (58.8%) were diagnosed with DED. There was no significant difference in spherical equivalent refraction between the DED and no-DED groups, but the lens opacity score was significantly higher in the no-DED group than in the DED group.

### 3.2. Comparison of Measurement Repeatability of Corneal Curvature Radius and Axial Length

Table 3 shows the results of the measurement repeatability for corneal curvature radius and axial length in the DED and no-DED groups. The absolute difference in the steep meridian was significantly larger in the DED group than in the no-DED group (*p* = 0.044). However, there were no significant differences in the absolute difference in the flat meridian and axial length between the DED and no-DED groups.

### 3.3. Correlation between DED Parameters and Measurement Repeatability

**Figure 1:**
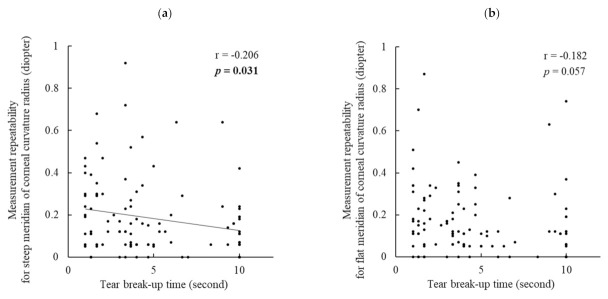

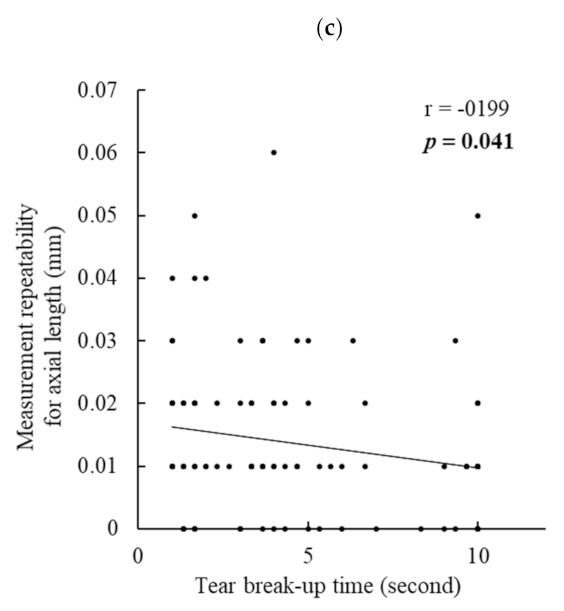
Correlations between tear break-up time and the measurement repeatability of corneal curvature radius and axial length: (**a**) steep meridian of corneal curvature radius, (**b**) flat meridian of corneal curvature radius, and (**c**) axial length. Tear break-up time was significantly correlated with the measurement repeatability of the steep meridian of corneal curvature radius and axial length but not with that of the flat meridian of corneal curvature radius. Boldface values indicate statistical significance. Boldface values indicate statistical significance. Correlation was evaluated using Pearson’s correlation analysis.

BUT had a significant negative correlation with the measurement repeatability of the steep meridian (r = −0.206, *p* = 0.031) (Figure 1a) and axial length (r = −0.199, *p* = 0.041) (Figure 1c), but it did not have a significant correlation with that of the flat meridian (r = −0.182, *p* = 0.057) (Figure 1b).

**Figure 2:**
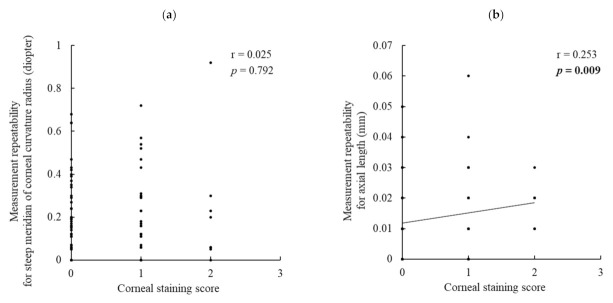
Correlations between the corneal staining score and measurement reproducibility of the steep meridian of corneal curvature radius and axial length: (**a**) steep meridian of corneal curvature radius and (**b**) axial length. The corneal staining score was significantly correlated with the measurement repeatability of axial length but not with that of the steep meridian of corneal curvature radius. Boldface values indicate statistical significance. Correlation was evaluated using Spearman’s correlation analysis.

The corneal staining score had a significant positive correlation with the measurement repeatability of axial length (r = 0.253, *p* = 0.009) (Figure 2b) but no correlation with that of the steep meridian (r = 0.025, *p* = 0.792) (Figure 2a).

Table 4 shows the results of stepwise multiple regression analysis. The results of this analysis indicated that BUT was more strongly associated with the measurement repeatability of axial length than with that of the corneal staining score.

## 4. Discussion

This study investigated the effect of DED on the repeatability of corneal curvature radius and axial length measurements in cataract patients. To this end, we first confirmed the high prevalence of DED (58.8%) in patients requiring cataract surgery. There have been two studies on the prevalence of DED in cataract patients scheduled for surgery in Japan. Amano et al. [15] and Miyake et al. [12] reported values of 37.8% and 69.7%, respectively, and our results were similar to the previously reported prevalence. These high values, including our results, strongly support the significance of investigating the impact of DED on cataract surgery.

In this study, the corneal curvature radius and axial length were measured using IOLMaster-500, which is currently one of the most widely used devices for biometric measurements of cataract patients [16]. The instrument measures the corneal curvature radius of the flat and steep meridians with an incorporated automatic keratometer; six spots of light are projected onto the cornea at a fixed angle of approximately 14°. The patterns of the reflected spots are captured by a camera, and the corneal curvature radius is calculated based on image separation [17]. In other words, the corneal curvature radius is calculated by capturing the light reflected from the air/tear film interface [18]. Since the tear film is unstable in patients with DED, light reflection may vary and lead to deterioration in measurement accuracy. This was the main reason we compared measurement repeatability of the corneal curvature radius between eyes with and without DED in this study.

Concerning the baseline characteristics of the patients in this study, the lens opacity score was slightly higher in the no-DED group than in the DED group (2.1 vs. 1.9), although there was no significant difference in spherical equivalent refraction between the groups. However, this effect on the measurements of corneal curvature radius was considered negligible because the crystalline lens did not affect the keratometric measurements. Additionally, it is unlikely that the lower score of the DED group negatively affected the measurement repeatability of axial length compared to the higher score of the no-DED group. As for DED parameters, BUT, the corneal staining score, and the conjunctival staining score were significantly worse in the DED group than in the no-DED group at baseline.

The absolute difference in the two assessments on the same day (measurement repeatability) for the steep meridian of the corneal curvature radius was significantly larger in the DED group than in the no-DED group. Additionally, measurement repeatability was significantly negatively correlated with BUT. That is, a shortened BUT caused larger errors in the corneal curvature measurements. This is the first study to clarify the relationship between BUT and the repeatability of corneal curvature measurements. As BUT is a principal index for unstable tear film, it can be rephrased that tear instability considerably affects the precision of corneal biometry. However, the corneal staining score showed no correlation with measurement repeatability. This indicates that the effect of corneal epithelial damage on keratometric measurements was not substantial.

In contrast, there was no significant difference in measurement repeatability of the flat meridian of the corneal curvature. Combined with the results of the steep meridian, it is deemed that the steep meridian was more susceptible to DED than the flat meridian. This is another novel finding of the present study. The reason for this is unclear, but the measurement principle of keratometry may have caused these results.

Regarding the measurement repeatability of axial length, no significant difference was observed between the DED and no-DED groups. However, interestingly, when analyzing all the patients’ data, the repeatability of axial length was significantly negatively correlated with BUT and the corneal staining score, and BUT showed a stronger contribution in the multiple regression analysis. These findings imply that not only keratometric readings but also axial length measurements can be influenced by DED. IOLMaster-500 generates infrared light with a wavelength of 780 nm to measure the axial length. This laser beam splits into two equal coaxial beams, which enter the eye and reflect at the interface of the cornea and retina, and the difference in frequency between the coaxial beams is detected by a photodetector [6,18]. Thus, there is a possibility that an unstable tear film and corneal epithelial damage somewhat affect the beam reflection at the corneal surface and decrease the measurement accuracy. However, we are unaware of the exact mechanism involved. Further studies should be conducted to confirm the current results and to clarify the underlying mechanism.

Epitropoulos et al. compared the repeatability of simulated keratometry between a hyperosmolar (i.e., DED) group and a normal (i.e., no DED) group and found that the percentage of eyes with a difference of more than 0.5 D was significantly higher in the hyperosmolar group [7], which is comparable to the results of our study. The authors suggested that DED with high tear osmolarity may induce unexpected postoperative refractive errors resulting from inaccurate preoperative keratometry in cataract surgery. Our findings support their suggestion with a new perspective of unstable tear film and epithelial damage of DED.

Recently, the ASCRS Cornea Clinical Committee reported a new consensus-based practical diagnostic ocular surface disease (OSD) algorithm to aid surgeons in efficiently diagnosing and treating visually significant OSD before any form of refractive surgery is performed [19]. The report emphasized that the importance of addressing OSD cannot be underestimated, because the presence of OSD potentially results in the following adverse outcomes: (1) unsatisfactory vision (e.g., refractive errors, fluctuating vision, and induced higher-order aberrations); (2) new or worsened OSD symptoms (e.g., foreign-body sensation, redness, and pain); and (3) postoperative infections, such as endophthalmitis. The committee also recommended postponing surgery and delaying the final refractive measurements until the OSD is fully treated and resolved. Our findings also support these statements.

This study has several limitations. First, this study was exploratory research with a small sample size, and data of both eyes were used if the diagnostic criteria were met. Further studies with a larger sample size that include only one eye for each patient are needed. Second, the corneal curvature radius and axial length were measured with the IOLMaster-500, which is not the latest instrument. The instrument was updated to the IOLMaster-700, which is based on swept-source optical coherence tomography. In addition, multiple instruments are used to measure the corneal curvature radius and axial length. Therefore, it is necessary to conduct further studies using other instruments and the latest devices. Third, the clinical significance of the reduction in measurement repeatability observed in this study has not been evaluated. The mechanism by which the decline in preoperative measurement repeatability due to tear instability affects postoperative outcomes should be investigated in another study.

## 5. Conclusions

The measurement repeatability of corneal curvature radius was decreased in eyes with DED compared to normal eyes. Shortened BUTs were related to a reduction in the measurement repeatability of corneal curvature radius and axial length.

## Figures and Tables

**Table 1:** Demographic data.

Demographic Data		Total (*N* = 69 Patients, 114 Eyes)
Sex	Male (%)	28 (40.6)
	Female (%)	41 (59.4)
Age (year)	Mean (SD)	73.4 (6.9)
	Median	75.0
	Min, Max	55, 84
	50 to 59 (%)	3 (4.3)
	60 to 69 (%)	19 (27.5)
	70 to 79 (%)	31 (44.9)
	80 or over (%)	16 (23.2)
Operative eye	Both eyes (%)	45 (65.2)
	One eye (%)	24 (34.8)

SD = standard deviation.

**Table 2:** Comparison of DED parameters, refraction, and lens opacity score between eyes with and without DED.

DED Examinations	DED		No DED		*p* Value
	Mean	SD	Mean	SD	
Tear break-up time (s)	2.66	1.28	8.06	2.63	<0.001 *
Corneal staining score	0.7	0.7	0.2	0.4	**<0.001 ^†^**
Conjunctival staining score	1.8	1.3	0.7	0.9	**<0.001 ^†^**
Spherical equivalent refraction (diopter)	−0.70	2.74	−1.36	2.91	0.251 *
Lens opacity score	1.9	0.6	2.1	0.6	**0.040 ^†^**

Lens opacities were observed with a slit-lamp microscope and scored using standard photographs based on the Emery–Little classification. DED = dry eye disease, SD = standard deviation. Boldface values indicate statistical significance. * DED vs. no DED: significant by Welch’s *t*-test. ^†^ DED vs. no DED: significant by Wilcoxon’s rank-sum test.

**Table 3:** Comparison of measurement repeatability of corneal curvature radius and axial length between eyes with and without DED.

			DED			No DED			*p* Value
			Mean	SD	Min, Max	Mean	SD	Min, Max	
Absolute difference between two measurements	Corneal curvature radius (diopter)	Steep meridian	0.21	0.19	0.00, 0.92	0.14	0.15	0.00, 0.64	**0.044**
		Flat meridian	0.18	0.16	0.00, 0.87	0.14	0.17	0.00, 0.74	0.219
	Axial length (mm)		0.015	0.013	0.00, 0.06	0.011	0.011	0.00, 0.05	0.147

Corneal curvature radius and axial length were assessed twice on the same day using the same instrument, and the absolute difference between the two assessments was calculated and used as an index of measurement repeatability. Corneal curvature radius was evaluated separately for the steep and flat meridians. DED = dry eye disease, SD = standard deviation. Boldface values indicate statistical significance. DED vs. no DED: significant by Welch’s *t*-test.

**Table 4:** Results of multiple regression analysis for evaluating the contributions of BUT and the corneal staining score to the measurement repeatability of axial length.

	Univariate Analysis				Multivariate Full Model					Multivariate Final Model Forward Selection Method (Stepwise)				
	RC	95% CI	*p* Value	Standard RC	RC	95% CI	*p* Value	Standard RC	VIF	RC	95% CI	*p* Value	Standard RC	VIF
BUT (s)	−0.0007	(−0.0014, 0.0000)	**0.041**	−0.1992	−0.0006	(−0.0013, 0.0001)	0.117	−0.1598	1.1125	−0.0007	(−0.0014, 0.0000)	**0.041**	−0.1992	1.0000
Corneal staining score	0.0033	(−0.0003, 0.0070)	0.073	0.1749	0.0024	(−0.0015, 0.0062)	0.223	0.1241	1.1125	-	-	-	-	-

Axial length was assessed twice on the same day using the same instrument, and the absolute difference between the two assessments was calculated and used as an index of measurement repeatability. BUT = tear break-up time, RC = regression coefficient, IC = confidence interval, VIF = variance inflation factor. Boldface values indicate statistical significance.

## Data Availability

The data presented in this study are available on request from the corresponding author.
